# Effects of Electroacupuncture on Ovarian Expression of the Androgen Receptor and Connexin 43 in Rats with Letrozole-Induced Polycystic Ovaries

**DOI:** 10.1155/2020/3608062

**Published:** 2020-07-14

**Authors:** Ge Xu, Andong Zhang, Jiandang Liu, Xi Wang, Jiwei Feng, Yuelai Chen

**Affiliations:** ^1^Shanghai University of Chinese Traditional Medicine, Shanghai 201203, China; ^2^Yueyang Hospital of Integrated Traditional Chinese and Western Medicine, Shanghai University of Chinese Medicine, Shanghai 200437, China

## Abstract

**Background:**

Polycystic ovarian syndrome (PCOS) occurs in women of reproductive age and is often characterized by reproductive and endocrine dysfunction. Androgens play a major role in PCOS, and previous studies reported abnormal expression of Connexin 43 (Cx43) in animal models of PCOS, suggesting an association of Cx43 with PCOS pathogenesis. Experimental and clinical evidence indicated that acupuncture may be a safe and effective approach for treating reproductive and endocrine disorders in women with PCOS. This study aimed to determine the effects of electroacupuncture (EA) on PCOS and its relationship with the expression of the androgen receptor (AR) and Cx43.

**Methods:**

In total, 30 female Sprague Dawley rats (6 weeks old) were randomly divided into three groups: control group, letrozole (LE) group, and LE + EA group. Rats were administered LE solution (1.0 mg/kg) for 21 consecutive days to induce PCOS. For the LE + EA group, additional EA treatment was conducted (2 Hz, 20 min/d) with “Guanyuan” (CV3) for 14 consecutive days. After hematoxylin-eosin staining, the ovarian structure was observed with an optical microscope, and serum levels of the following hormones were examined via enzyme-linked immunosorbent assay (ELISA): testosterone (T), estradiol (E_2_), sex hormone-binding globulin (SHBG), follicle-stimulating hormone (FSH); luteinizing hormone (LH), insulin (INS), anti-Müllerian hormone (AMH), and inhibin B (INHB). Fasting blood glucose (FBG) levels were evaluated using glucose oxidase-peroxidase. Ovarian mRNA and protein expressions of AR and Cx43 were determined by real-time RT-PCR and Western blot analysis.

**Results:**

EA was found to restore the cyclicity and ovarian morphology in the PCOS rat model. Serum derived from the LE + EA group showed significant decreases in the levels of T, free androgen index (FAI), LH, LH/FSH ratio, AMH, INHB, and fasting serum insulin (FINS), and significant increases in the levels of E_2_, FSH, and SHBG. Western blot analysis showed a decreased protein expression of ovarian AR and Cx43; real-time RT-PCR showed reduced expression of ovarian mRNA levels of AR and Cx43.

**Conclusions:**

In conclusion, our results showed that EA can ease hyperandrogenism and polycystic ovary morphology in PCOS rats. Furthermore, EA counteracted the letrozole-induced upregulation of AR and Cx43. These results suggested that acupuncture can break the vicious cycle initiated by excessive androgen secretion and may be an effective treatment method for improving the reproductive and endocrine dysfunction caused by PCOS.

## 1. Background

Polycystic ovarian syndrome (PCOS) is a group of syndromes with multiple pathogenesis and clinical polymorphism and an endocrine and metabolic disorder in women. Reported PCOS incidence ranges from 6% to 20% in different areas, depending on the criteria used [1–4]. Its phenotypic expression varies and is characterized by ovulatory dysfunction, infertility, hirsutism, and obesity [5]. Histological analyses have shown numerous antral follicles in PCOS patients. However, the etiology of this heterogeneous condition remains unknown. However, there is evidence that human PCOS is associated with hyperandrogenism, hyperinsulinemia, and insulin resistance (IR), hypothalamus-pituitary-ovarian axis dysfunction, and progression to type II diabetes [6, 7]. The proliferation of follicular thecal cells and overproduction of androgens are the primary causes of the pathological manifestations of PCOS [8].

Studies have shown that acupuncture may be a safe and effective way to treat reproductive endocrine dysfunction in women with PCOS [9]. At present, there are many clinical reports on acupuncture treatment of PCOS, and most of these studies confirmed the effect of acupuncture, reporting that acupuncture can improve at least one or several symptoms or indicators in PCOS [10]. Acupuncture can improve the egg quality of women with PCOS undergoing *in vitro* fertilization and embryo transfer (IVF-ET) and improve the clinical pregnancy rate of IVF [11]. However, it is also believed that acupuncture has no obvious effect on PCOS [12].

In several studies, it has been shown that repeated low-frequency EA rehabilitated estrous cyclicity and regulated gonadotropin-releasing hormone and AR expression in the hypothalamus of rats, regulated u, К receptor mRNA expression, and lowered testosterone levels, while manual stimulation can reduce estrogen, progesterone, and kisspeptin receptor expression [13, 14]. EA regulated circulating gonadotropin levels in PCOS mice, independent of the effects of sex hormones or *β*-epinephrine, and affected the ovarian adiponectin system [15]. In rats with estradiol valerate-induced PCOS, the concentration of *β*-endorphins in the hypothalamus and plasma was significantly lower than that found in control rats, and treatment with EA significantly increased the concentration of *β*-endorphins in the hypothalamus [16]. In a previous study, it was found that EA could improve the local androgen excess environment in the ovaries by regulating protein and mRNA expression of P450arom and P450c17*α* in the ovarian tissue of rats with PCOS and by improving the reproductive endocrine and metabolic disorders associated with PCOS [17]. Compared with the physiotherapy group, 10–13 weeks of EA intervention improved the ovulation frequency of women with PCOS and regulated serum sex hormone levels [18].

The prevalence of IR and hyperinsulinemia in PCOS patients is 50%–70% [19]. This prevalence is higher in overweight women and reaches 95% [20]. Acupuncture may improve IR by increasing the number and affinity of insulin receptors in obese individuals. In addition, it has been shown that EA does not rely on insulin to stimulate glucose conversion in skeletal muscles and enhances insulin sensitivity during glucose conversion in rats. Furthermore, EA has been reported to restore the expression of leptin and uncoupling protein 2 and to increase plasma levels of insulin-like growth factor-1 [21–24].

Androgen plays its role by binding to the AR. The AR is widely expressed in granulosa cells at the early stage of follicular development, and ARs are abundant in preantral follicles [25]. With the development of follicles, AR expression in granulosa cells gradually decreases and reaches a minimum level before ovulation [26]. The differential expression of AR at different times ultimately affects the follicular outcomes by coregulating multiple mechanisms inside and outside the ovary.

In mice, most follicles in the ovaries of mice lacking Connexin 43 (Cx43) stop developing before granulosa cell stratification, resulting in an abnormal follicular structure, vacuolization of oocytes and granulosa cells to a certain extent, inability to resume oocyte meiosis, and the inability of the oocyte to be fertilized [27, 28]. This is mainly because the lack of Cx43 results in the inability to establish communication, material exchange, and signal transduction between granulosa cells, and eventually failure of granulosa cell proliferation and stratification. In addition, in several studies, it was found that Cx43 can resist granulosa cell apoptosis, promote follicular selection and recruitment, and its expression intensity is negatively correlated with the apoptotic index of granulosa cells [29, 30]. Cx43 plays an important role in the process of oocyte meiosis and follicular selection. In recent years, studies have found abnormal expression of Cx43 in PCOS animal models, suggesting that Cx43 may be associated with PCOS pathogenesis [31]. In testosterone-treated mouse ovaries, the location and quantity of AR and Cx43 were significantly changed, with the expression of AR significantly being increased, whereas the expression of Cx43 was significantly decreased [32]. However, in another study, it was shown that the expression of Cx43 protein was increased in androstenedione-induced polycystic ovaries of rats and androstenedione upregulated Cx43 protein levels in granulosa cells *in vitro* [33]. In addition, it was found that the AR pathway plays an important role in the regulation of Cx43 expression in prostate cancer cells. AR may be the upstream signal of Cx43 [34]. However, it is not clear whether EA has any effect.

Previous studies have demonstrated that letrozole-induced PCOS models have characteristics similar to those of human patients, such as hyperandrogenism, LH hypersecretion, follicular dysplasia, and anovulation [35]. In our previous study, we compared the efficacy of different acupoints and selected the one with the best therapeutic effects on hyperandrogenism among four groups (ST36, SP6, CV3, and comprehensive groups) [36]. In this study, we attempted to gain a deeper understanding of the effects of EA on PCOS and its relationship with AR and Cx43 expression using a letrozole-induced PCOS model in rats.

## 2. Methods

### 2.1. Animals and Experimental Procedure

In this study, 30 female Sprague Dawley rats (6 weeks old) were housed in a controlled temperature environment (20–23°C), humidity (40%–60%), and 12 h light : 12 h dark cyclical alternates with ad libitum availability of food and water. All procedures described here were approved by the Animal Ethical Committee of Shanghai University of Chinese Traditional Medicine (Shanghai, China).

Thirty rats with comparable weights (160 ± 20 g) were randomly assigned to three different groups: control group, LE group, and LE group receiving EA (LE + EA group). The PCOS model was established by administering (via gavage) the animals letrozole solution (1.0 mg/kg) once daily for 21 consecutive days. The control group was administered 1% CMC (10 mg/kg) by gavage. During modeling, rats were weighed using a JA 31002 electronic balance (Shanghai Jing Tian Electronic Instrument Co., Ltd., Shanghai, China). From the second day after modeling, the LE + EA group underwent EA treatment for 14 consecutive days. The control and LE groups were not given any treatment, except for fixation.

### 2.2. Estrus Cyclicity Was Observed Using a Microscope

Estrus cyclicity was monitored daily at 9 AM from the first day after the first administration to the 36^th^ consecutive day by microscopic observation of the type of epithelial cells in the vaginal smear. Vaginal cells were collected via saline lavage, fixed with methanol, and stained with Pap stain. Animals with regular 4-5-day cycles, including proestrus, estrus, postestrus, and diestrus, were defined as normal cyclic rats, whereas those whose estrus cycles stopped for four consecutive days in diestrus phases were termed acyclic rats.

### 2.3. EA Treatment

In the LE + EA group, acupoints CV-3 (on the ventral midline at approximately the upper 3/5 and lower 2/5 of the line) and the point 5 mm next to CV-3 at the same horizontal axis were used. We inserted 25 mm acupuncture needles (*ϕ* 0.22) to a depth of 0.3–0.5 cm and attached them bilaterally to the Hwato SDZ-II electroacupuncture therapeutic apparatus (Suzhou medical Appliance factory, Suzhou, China). The points were electrically stimulated with a low frequency of 2 Hz, thereby adjusting the intensity to 2 mA. The retention time was 20 min. Rats were treated daily for 14 consecutive days.

### 2.4. Blood and Tissue Collection

On the day after the last EA treatment, the vaginal smears were examined before sampling. We collected blood samples and ovarian tissue samples on the diestrus stage of the estrus cycle and between 8 and 11 AM. Rats that were not on diestrus that day were sampled on a different day when they entered diestrus. Rats were anesthetized with 3% pentobarbital sodium (0.1 ml/100 g of body weight), and then blood samples were obtained from the abdominal aorta after overnight fasting. Blood samples were then centrifuged at 3000*g* for 15 min at 4°C, and serum was stored at −80°C. Subsequently, ovarian dissection was performed rapidly on ice. The wet weight of the dissected ovaries was measured using BS 124S electronic balance (Sartorius, Göttingen, Germany); from each rat, the left ovary was fixed in 4% paraformaldehyde for histological examination, and right ovary was stored at −80°C until extraction.

### 2.5. Morphological Analysis

From each rat, ovarian tissue was collected after blood collection, fixed in 4% paraformaldehyde, blocked in paraffin, and cut into serial 4 *μ*m sections. Sections were stained with hematoxylin-eosin and examined using a Leica DM 2000 microscope (Leica, Wetzlar, Germany).

### 2.6. Blood Sample Assays

Serum concentrations of T, E_2_, FSH, LH, SHBG, AMH, INHB, and FINS were quantified using rat ELISA Kits (Abcam Systems, Cambridge, UK). All experiments were performed following the manufacturer's instructions. The detection limit was 1.0 pg/mL for T, 0.1 pmol/L for E_2_, 0.1 IU/L for FSH, 0.1 mIU/L for LH, 0.1 nmol/L for SHBG, 10 pg/mL for AMH and INHB, and 0.1 mU/L for FINS. For all ELISA Kits, the intra- and interassay variations were 10% and 15%. FBG levels were determined using a glucose oxidase kit (Rongsheng Biotech, Shanghai, China).

### 2.7. RNA Isolation and Real-Time RT-PCR

Total RNA was isolated using TRIsoln reagent (Invitrogen, Carlsbad, CA, USA), and total RNA (2 *μ*g) was reverse transcribed using the Superscript Reverse Transcriptase kit (Takara, Japan). Further, real-time PCR was performed with a TB Green™ Premix Ex Taq™ (Takara, Japan) using an ABI 7900 real-time PCR system and SDS software (Applied Biosystems, Foster City, CA, USA). Primers were procured from Sangon (Sangon Biotech, Shanghai, China) and sequences are shown in Table 1. The mRNA expression values for each sample were calculated following the ^ΔΔ^Cq method.

### 2.8. Western Blot Analysis

We homogenized each group of rat ovaries in ice-cold RIPA Lysis Buffer (Beyotime, Shanghai, China). Then, sample homogenates were incubated on ice for 30 min and centrifuged (13200*g*) for 15 min at 4°C. The supernatants were collected, and protein concentration was determined using the BCA Protein Assay Kit (Beyotime, Shanghai, China) and stored at −80°C until electrophoresis. The samples were denatured by adding the sample buffer (1.0 mol/L Tris-HCl (pH 6.8; 0.6 ml), 50% glycerol (5 mL), 10% SDS (2 mL), *β*-mercaptoethanol (0.5 ml), 1% bromophenol blue (1 mL), and deionized water to make the total volume to 10 mL). Total protein (30 *μ*g) was separated on 10% SDS-PAGE gels in Tris-glycine and 0.1% SDS buffer and transferred to a nitrocellulose sheet in 25 mm Tris, 192 mm glycine, and 20% methanol buffer at 200 mA for 2 h. Membranes were blocked for 2 h at room temperature in 5% nonfat dry milk and incubated overnight at 4°C with an anti-androgen receptor antibody (Abcam, Cambridge, UK) or anti-Cx43 antibody (Abcam, Cambridge, UK) at 1/200 or 1/1000 dilutions, respectively. Then, imprints were washed and incubated for 2 h at room temperature with a secondary antibody bound to horseradish peroxidase (HRP) (1 : 5000, Abcam, Cambridge, UK). The protein-antibody complexes were then visualized using ECL Plus Western Blot Detection Reagents (GE Healthcare, Pittsburgh, PA, USA) and photographed with Clinx ChemiScope Mini Series Western Blot Imaging System (CLINX Science Instruments, Shanghai, China). Protein signals were quantified using the ImageJ software. *β*-Actin or Gapdh served as a loading control and an internal reference.

### 2.9. Statistical Analysis

Experimental data are shown as the mean ± SEM, and statistical analyses were performed using the SPSS software package (SPSS, version 20.0; Chicago, IL, USA). Differences between groups were evaluated using one-way analysis of variance (ANOVA). The LSD test was used for pairwise comparison of homogeneity of variance, and Dunnet's test was used for heterogeneity of variance. *P* < 0.05 was considered significant.

## 3. Results

### 3.1. Improvement in Estrous Cyclicity

After modeling, rats administered letrozole displayed abnormal estrous cycles with estrus disappearing on approximately the 12th day and remained in the diestrus stage. All rats in the control group showed normal estrous cyclicity. Thus, the model animal was successfully established. In the LE + EA group, 7 of 10 rats resumed estrous cyclicity, and epithelial keratinocytes in vaginal smear were observed under a microscope during treatment. The remaining 3 rats in this group showed no response to EA treatment until the time of terminal kill. The 10 rats in the LE group were still in the diestrus stage at the time of the terminal kill.

### 3.2. Rat Body Weight and Ovary Weight

**Figure 1:**
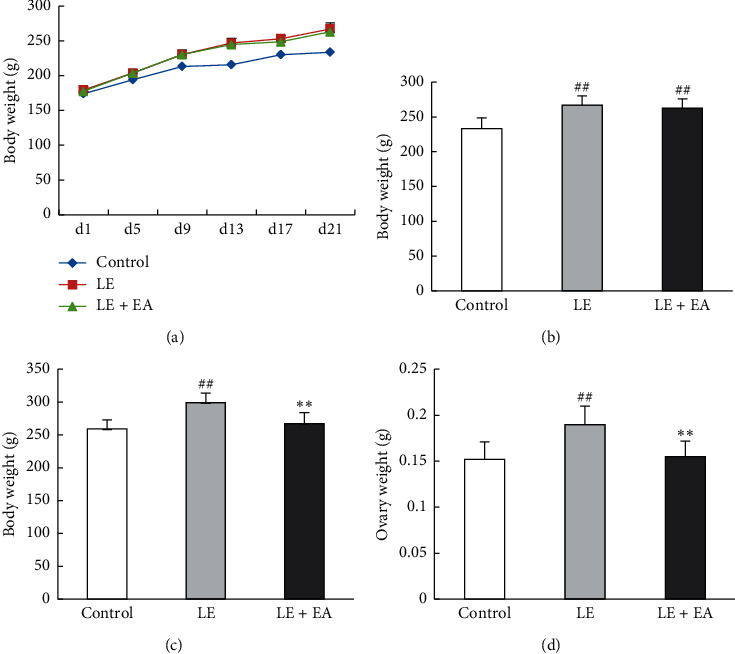
Body weight and ovarian weight of rats. (a) Changes in body weight during modeling. (b) Pretreatment body weight (on the 21st day). (c) Posttreatment body weight. (d) Ovary weight. Error bars represent the SEM. *n* = 10 per group. #*P* < 0.05; ##*P* < 0.01 vs control group; *∗∗P* < 0.01 vs LE group.

No significant differences were observed in the initial body weight between groups. From the 5th day after modeling to the end of modeling, the increase in body weight of rats in each modeling group was significantly faster when compared to that in the control group (Figure 1(a)), and the body weight was higher in the modeling groups compared to that in the control group at the end of modeling (*P* < 0.01; Figure 1(b)). This conforms to the characteristics of PCOS model rats. Following EA treatment, the body weight of rats in the LE + EA group was reduced compared with that of rats in the LE group (*P* < 0.05; Figure 1(c)).

The ovarian weight of rats in the LE group significantly increased compared with that of rats in the control group (*P* < 0.01). In addition, the ovarian weight of rats in the LE + EA group decreased significantly when compared with that of rats in the LE group (*P* < 0.01) (Figure 1(d)).

### 3.3. Ovarian Morphological Changes

In the control group, corpus luteum and follicles at different stages of development were observed. Granulosa cells were arranged in multiple layers within follicles. In the LE group, the surface of the ovary was pale and the capsule was thickened, with cystic follicles protruding. Microscopically, the number of granulosa cell layers in the LE group sections was less, and oocytes and radiating crowns disappeared in the cystic follicles.

**Figure 2:**
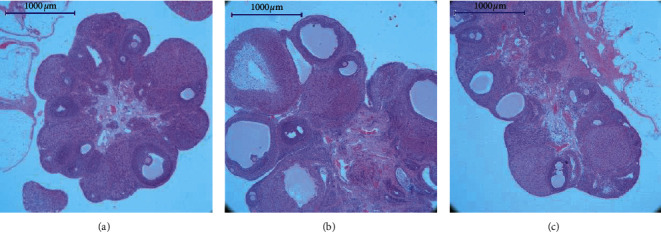
Histology of ovaries. Ovaries were stained with hematoxylin and eosin: (a) control group; (b) LE group; (c) LE + EA group; magnification ×400. Scale bars represent 1000 *μ*m in each case.

Compared with the LE group, the LE + EA group showed a decrease in ovary size as well as a decrease in the number of cystic follicles. Furthermore, an increase in the granulosa cell layer of follicles was observed in the LE + EA group, and submature follicles with cumulus were observed (Figure 2).

### 3.4. Circulating Hormone Level

Testosterone levels were significantly elevated in the LE group (*P* < 0.01) with a decrease in SHBG levels (*P* < 0.01), resulting in a higher FAI (*P* < 0.01) in rats with PCOS compared with the control group. After EA treatment, levels of circulating testosterone were lower, and SHBG levels were higher in the LE + EA group compared to the LE group, whereas FAI was lower in LE + EA group compared to the LE group (*P* < 0.01).

Estradiol levels were significantly decreased in the LE group (*P* < 0.01) compared with the control group. After EA treatment, levels of circulating estradiol were higher in LE + EA group compared to the LE group (*P* < 0.01).

**Figure 3:**
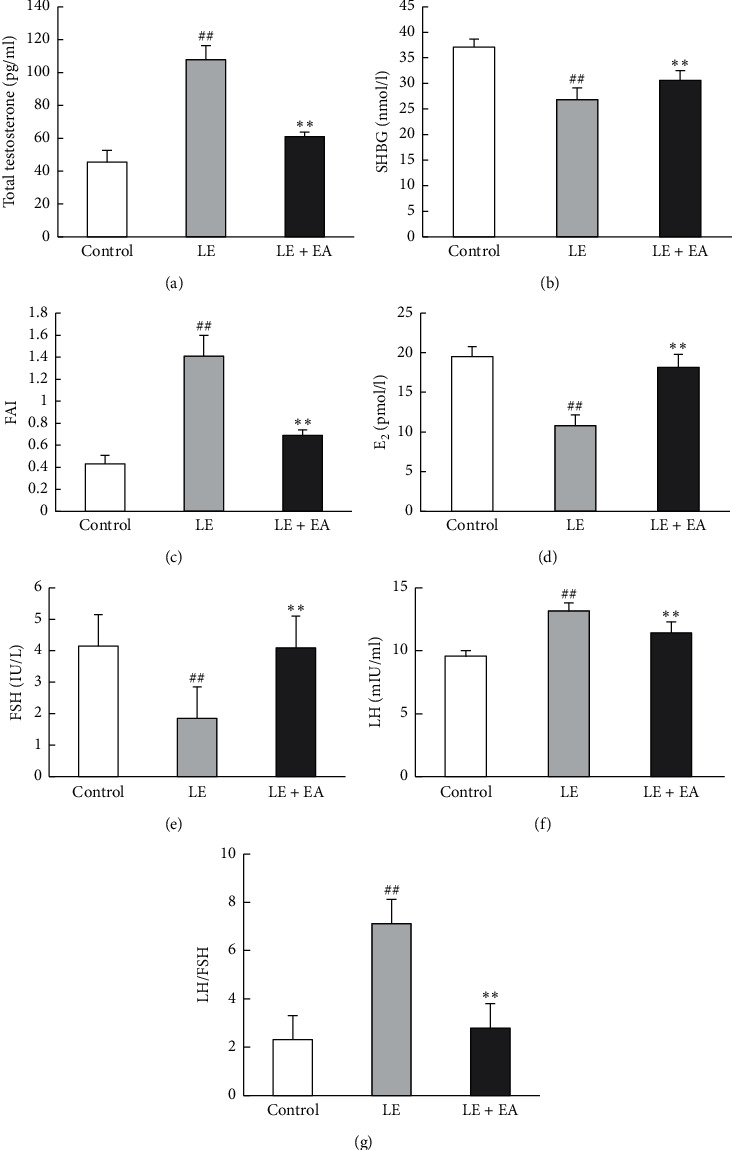
(a) Serum levels of testosterone T, (b) sex hormone-binding globin (SHBG), (c) fasting serum insulin (FAI), (d) estradiol (E_2_), (e) follicle-stimulating hormone (FSH), (f) luteinizing hormone (LH), and (g) LH/FSH in rats. Error bars represent SEM, *n* = 10 per group. ##*P* < 0.01 vs control group; *∗∗P* < 0.01 vs LE group.

Levels of circulating LH were higher and FSH levels were lower in the LE group compared with those in the control group (*P* < 0.01, *P* < 0.01), resulting in a higher LH/FSH ratio (*P* < 0.01) in rats with PCOS. EA treatment decreased LH (*P* < 0.01) and increased FSH in the LE + EA group (*P* < 0.01). Furthermore, the LH/FSH ratio was lower in the LE + EA group compared to the LE group (*P* < 0.01) (Figure 3).

### 3.5. Circulating Levels of AMH and INHB

**Figure 4:**
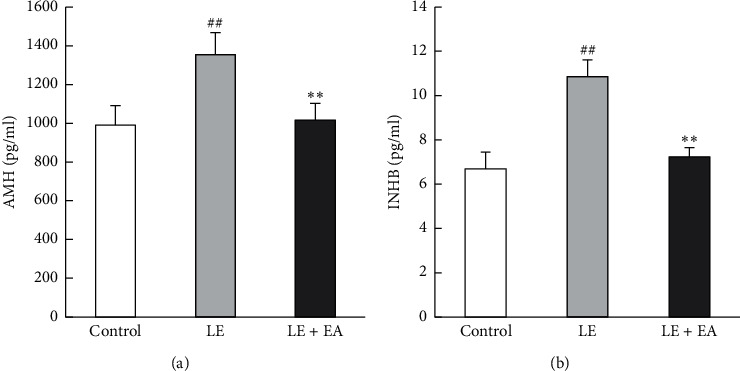
(a) Serum levels of anti-Müllerian hormone (AMH) and (b) inhibin B (INHB) in rats. Error bars represent the SEM, *n* = 10 per group. ##*P* < 0.01 vs control group; *∗∗P* < 0.01 vs LE group.

Levels of circulating AMH and INHB were increased in the LE group (*P* < 0.01) compared with the control group, and EA treatment decreased AMH and INHB levels in the LE + EA group (*P* < 0.01) (Figure 4).

### 3.6. Levels of FINS and FBG and Homeostasis Model-Insulin Resistance (HOMA-IR)

**Figure 5:**
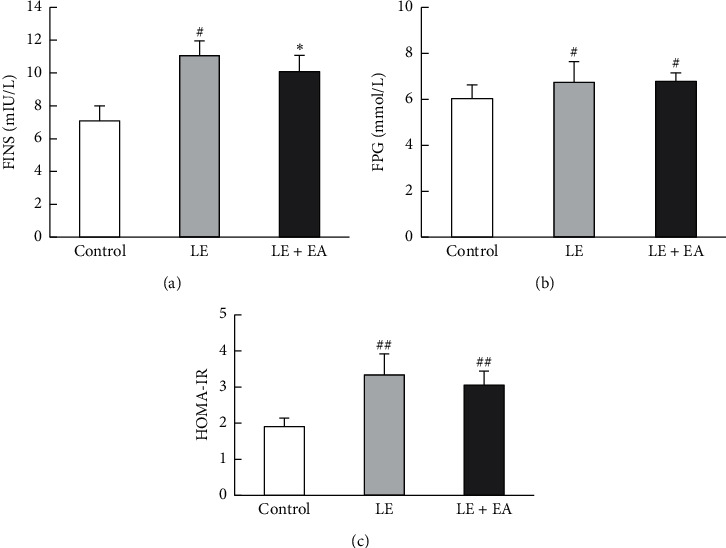
(a) Levels of free androgen index (FINS), (b) fasting blood glucose (FBG), and (c) HOMA-IR in rats. Error bars represent the SEM, *n* = 10 per group. #*P* < 0.05, ##*P* < 0.01 vs control group; *∗P* < 0.05 vs LE group.

Compared with the control group, the LE group showed increases in FINS, FBG, and HOMA-IR (*P* < 0.05, *P* < 0.01). After EA treatment, levels of circulating insulin were decreased in the LE + EA group compared with the LE group (*P* < 0.05). In addition, FBG levels were similar between LE and LE + EA groups (6.74 ± 0.91 mmol/L, 6.79 ± 0.36 mmol/L). In addition, HOMA-IR tended to be lower in the LE + EA group; however, the difference was not significant (Figure 5).

### 3.7. Immunolocalization and Expression of AR and Cx43 in Control Rat Ovary

**Figure 6:**
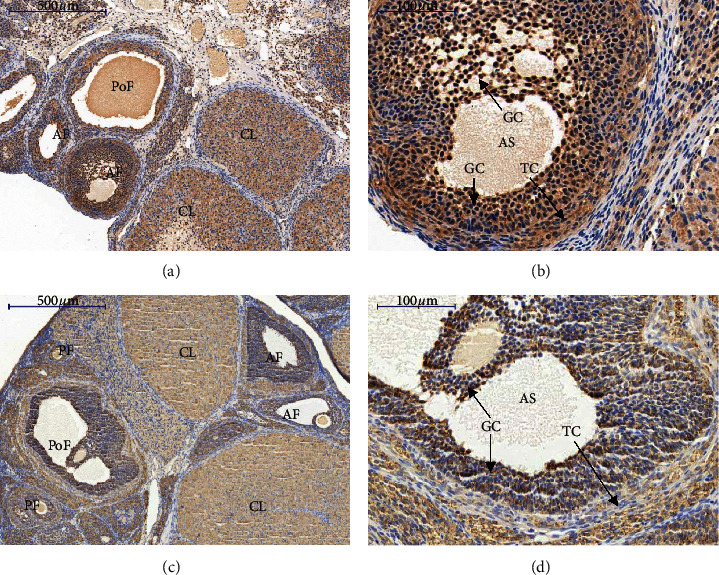
Immunolocalization and expression of the androgen receptor and Connexin43 in the ovary of a control rat. (a-b) Expression and localization of the androgen receptor (AR) in control rat follicles. Positive AR immunostaining was mainly observed in the nuclei of cells at the different developmental stages and corpora lutea (a). Positive AR immunostaining was observed in the nuclei of granulosa cells and theca cells in the antral follicle (b). (c-d) Expression and localization of Cx43 in follicles of control rats. Cx43 was observed as punctuate staining on the borders of granulosa cells in preantral follicles, large antral follicles, preovulatory follicles, and corpora lutea (c). Cx43 displayed punctate to the linear expression on the borders of granulosa cells, between granulosa cells and theca cells, as well as between granulosa cells and oocyte in the preovulatory follicle of control ovary (d). The expression was shown in brown, and the nuclei were stained in blue. PoF, preovulatory follicle; AF, antral follicle; AS, antral space; PF, primary follicle; TC, theca cell; GC, granulosa cell; CL, corpus luteum. Scale bars represent 500 *μ*m in (a) and (c). Scale bars represent 100 *μ*m in (b) and (d).

To investigate the localization and expressions of AR and Cx43 in the rat ovary, immunofluorescence was performed on samples in the control group. As shown in Figure 6, AR was expressed in the nuclei of granulosa cells and theca cells (TCs) in ovarian follicles at every developmental stage, with the highest expression levels found in granulosa cells (GCs) of preantral and small antral follicles. The expression of AR decreased when follicles entered the preovulation stage. In addition, Cx43 was expressed on the borders of GCs of small and large follicles and the corpus luteum (CL). Cx43 was observed between granulosa cells as well as between granulosa cells and oocytes in large antral follicles and preovulatory follicles. Cx43 was hardly detected at the thecal cell layer.

### 3.8. Ovarian mRNA Expression of AR and Cx43

**Figure 7:**
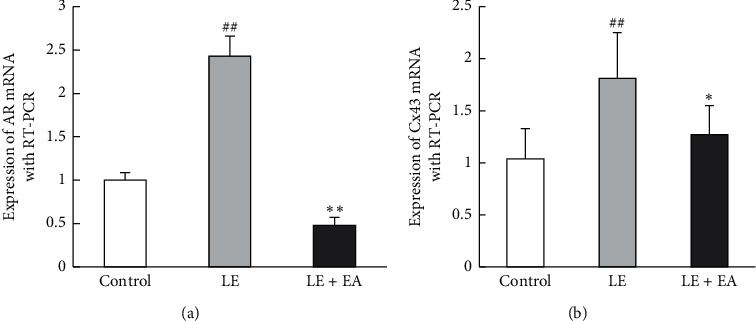
(a) Effects of EA on mRNA expression levels of AR and (b) Cx43 mRNA in ovarian tissues of rats in the three groups. Values are shown as levels of expression relative to that of Gapdh. Error bars represent the SEM, *n* = 5 rats/group, ##*P* < 0.01; #*P* < 0.05 vs control group; *∗∗P* < 0.01; *∗P* < 0.05 vs LE group.

Ovarian mRNA expression of AR and Cx43 was higher in the LE group compared to the control group (*P* < 0.01). In addition, the mRNA expression of AR and Cx43 was lower in the LE + EA group compared to the LE group (*P* < 0.01, *P* < 0.05) (Figure 7).

### 3.9. Ovarian Protein Expressions of AR and Cx43

**Figure 8:**
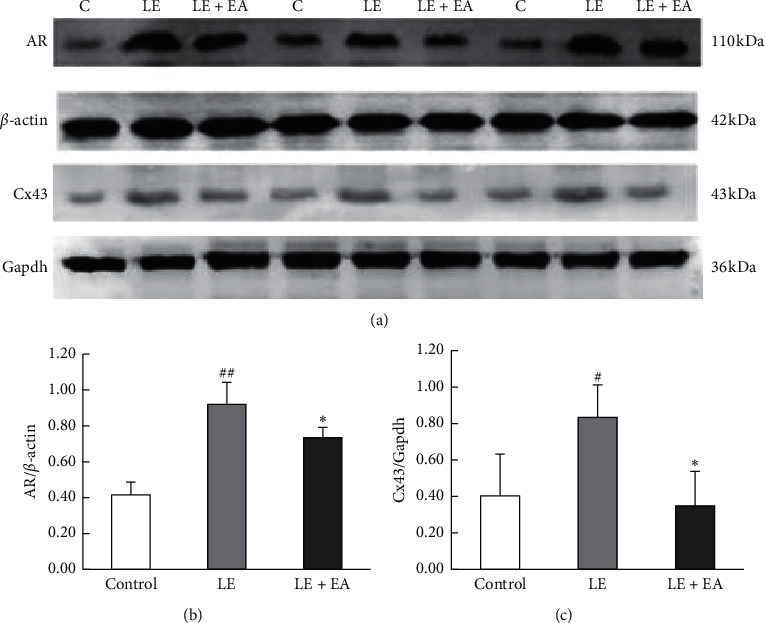
The effects of EA on AR and Cx43 expression in ovarian tissues of rats in the three groups (*n* = 3 rats/group). (a) Representative immunoblots of AR and Cx43 protein are shown. (b) The bar graph shows the levels of expression relative to that of actin bands in rats' ovarian tissue from three groups. Error bars represent the SEM. ##*P* < 0.01 vs control group; *∗P* < 0.05 vs LE group. (c) The bar graph shows the levels of expression relative to that of Gapdh bands in rats' ovarian tissue from three groups. Error bars represent the SEM. #*P* < 0.05 vs control group; *∗P* < 0.05 vs LE group.

Protein expressions of AR and Cx43 in ovaries were higher in the LE group compared to the control group (*P* < 0.05). In addition, protein levels of AR and Cx43 were lower in the LE + EA group compared to the LE group (*P* < 0.05) (Figure 8).

## 4. Discussion

Hyperandrogenism is the basic pathological feature of PCOS and plays an important role in the pathogenesis of PCOS. Androgen excess can lead to a range of clinical symptoms: amenorrhea, hirsutism, acne, and alopecia [37]. In women with PCOS, excess androgens are mainly from the ovaries, and excess androgen synthesis in the ovaries is associated with multiple factors, which may be affected by extraovarian factors such as LH, insulin, neuroendocrine changes, and local factors within the ovary such as INHB and AMH.

Increased expressions of androgen synthesis catalytic enzymes such as cytochrome P45017*α*hydroxylase and cytochrome P450 cholesterol side-chain lyase seem to be directly responsible for increased androgen production [38]. Hyperandrogenemia can cause hypothalamus desensitization to the negative feedback of progesterone/estrogen, thereby further increasing gonadotropin secretion, and increasing the production of ovarian androgens, causing a self-driven vicious cycle. In addition, androgens directly impair follicular development and the formation of dominant follicles, thus leading to the formation of polycystic ovarian morphology [39]. In addition to androgens, IR also affected hypothalamic sensitivity to the negative progesterone feedback, leading to increased gonadotropin secretion. However, the mechanism underlying this effect is unclear [40, 41]. Hyperinsulinemia can reduce the production of SHBG in the liver and decrease the circulating SHBG levels, thus increasing the level of bioavailable free androgens [42].

In recent studies, it was shown that AMH and INHB were two major ovarian local regulators involved in the pathogenesis of PCOS [43, 44]. INHB is the major inhibitory factor of FSH secretion and may be involved in the hyporegulation of late follicular FSH. INHB can also increase the synthesis of androgens in follicular thecal cells. It has been shown that INHB can directly promote the expression of P450c17*α* and the synthesis of androgens [45]. AMH can reduce the sensitivity of antral follicles to FSH, thereby inhibiting the growth of preantral follicles and small antral follicles. Moreover, AMH can reduce the synthesis of E_2_ in granulosa cells by inhibiting the activity of aromatase in the ovary, which results in androgen accumulation. In previous studies, it has been demonstrated that the AMH concentration in serum and follicular fluid in women with PCOS accompanied by hyperandrogenemia was higher compared to that in women with normal androgen levels. These findings showed that AMH is positively correlated with androgen [46, 47].

In our study, we successfully established a rat model of PCOS with the characteristics of polycystic ovaries, obesity, irregular estrous cyclicity, hyperandrogenism, increased plasma insulin levels, decreased plasma SHBG levels, and increased LH concentrations. In addition, we showed that EA could restore the estrous cyclicity and ovary morphology, reduce the body mass of PCOS rats, downregulate plasma levels of T, FAI, LH, and FINS, and upregulate plasma levels of SHBG, E_2_, and FSH. This animal model also exhibited a significant increase in INHB and AMH. Simultaneously, when compared with the LE group, the LE + EA group showed lower INHB and AMH levels. These findings indicated that EA not only directly reduced the expression of androgen synthesis enzymes [17] but also had a benign effect on the factors affecting hyperandrogenism. Increases in blood glucose level, insulin level, and insulin resistance index in PCOS rats indicated that letrozole-induced PCOS rats showed the characteristics of metabolic disorders. EA treatment was found to reduce the insulin level in PCOS rats, had a tendency to reduce insulin resistance index, but had no significant effect on blood glucose levels. A possible explanation for the nonsignificant effect of EA on metabolic regulation may be related to acupoint selection, as CV-3 is advocated in TCM theory to mainly regulate reproductive dysfunction.

Androgens activated the AR, a key transcription factor mediating androgen-induced signaling as well as a member of the nuclear receptor. AR-mediated androgen actions played an important role in follicular development. However, it acts as a double-edged sword, and in addition to the positive effects of androgen on follicular development, abnormal androgen levels, especially those found in hyperandrogenism, seriously suppress normal follicular development, and the most typical evidence comes from PCOS [48]. The AR has been shown to be present in all stages of follicular development, with the highest expression levels found in GCs of preantral and small antral follicles [25, 49]. The expression of AR was found to gradually decrease when follicles entered the preovulation stage [32].

Gap junction protein (Connexin, Cx) is the main component of the gap junctions. In ovaries, Cx43, encoded by Gja1, has been found to be the main Connexin expressed in developing follicles forming the gap junctions coupling granulosa cells [50]. Cx43 is essential to ensure normal proliferation and stratification of granulosa cells and to maintain normal follicular structure and development. Cx43 knockout mice had folliculogenesis arrest in their primary stage and developed incompetent oocytes [27]. Regulation of the expression of GJIC and Connexin protein by steroids has been documented in ovarian cells [51].

Considering the importance of GJIC for GC differentiation and oocyte growth, in addition to the important role of steroids in controlling GJIC, the current study was designed to study the effect of EA on the expression of AR and Cx43 in PCOS rat ovary. In this study, the expression of AR and Cx43 was significantly increased following letrozole administration. This was consistent with the results presented in previous studies, which demonstrate that dihydrotestosterone or androstenedione increased the expression of Cx43 in ovarian tissues *in vivo* or *in vitro* [33, 52]. However, in a previous study, it was found that AR expression was significantly increased, following testosterone administration (injected intraperitoneally with testosterone at a dose of 1.3 mg/kg for 7 days), and positive Cx43 immunostaining was notably weakened in the whole ovary. In preovulatory follicles, Cx43 expression markedly decreased between cumulus granulosa cells and oocytes, whereas Cx43 expression almost disappeared between granulosa cells as well as between granulosa cells and theca cells [32]. One plausible explanation for this discrepancy may be that different modeling methods and dosages regulated Cx43 expression differentially. In our study, the increased expression of Cx43 may be associated with the increased number of preantral and small antral follicles in the whole ovary [53]. This partially validated previous findings, demonstrating that Cx43 had the ability to resist granulosa cell apoptosis [29, 30]. Furthermore, EA treatment decreased the expression of AR, thereby suggesting that EA not only affected the secretion of androgen but also regulated the expression of AR. In addition, EA reversed letrozole-induced Cx43 increase. The results obtained in the present study did not fully support the hypothesis of direct regulation of Cx43 by androgens but suggested an association between the AR and Cx43 expression.

Based on the theory of traditional Chinese medicine and experimental studies, we selected acupoints with better therapeutic effects to comprehensively evaluate the therapeutic effect of EA on PCOS rats. The results confirmed the effect of EA on adjusting hormone levels and polycystic ovary morphology in PCOS rats as well as regulating ovarian local factors such as AMH, INHB, AR, or Cx43. In this work, we demonstrated for the first time the EA modulation of Cx43 in letrozole-induced PCOS rats. The limitation of our study was that the underlying mechanism of interaction between AR and Cx43 expression and the role of EA were not discussed. Therefore, further studies will be necessary to clarify the underlying mechanism(s) involved.

## 5. Conclusions

In conclusion, our findings indicated the effectiveness of EA in restoring the estrous cyclicity and ovary morphology and regulating circulating sex hormone levels and hyperinsulinemia in rats with letrozole-induced PCOS. Furthermore, EA normalized the letrozole-induced upregulation of AR and Cx43. Taken together, these results suggested that acupuncture may be an effective treatment method to improve the reproductive and endocrine dysfunction resulting from PCOS and could break the vicious cycle initiated by excessive androgen secretion. Guan yuan was the first acupoint to be considered in the treatment of PCOS.

## Figures and Tables

**Table 1:** Primer sequences of targeted genes in rats.

Targeted genes	Forward and reverse primers	Amplification size (bp)	Accession number
AR	TTTTGAGTTTTGTTGTATT	173	NM_012502
	CTCTCTCTGTTTGTTTCTT		

Cx43	CCACTCTCGCCTATGTCTCC	110	NM_012567
	TAGTTCGCCCAGTTTTGCTC		

Gapdh	TCCTGCACCACCAACTGCTTAG	102	NM_017008
	AGTGGCAGTGATGGCATGGACT		

## Data Availability

The datasets used and/or analyzed in the current study are available from the corresponding author upon reasonable request.

## Abbreviations

PCOS: Polycystic ovarian syndrome
T: Testosterone
E_2_: Estradiol
FSH: Follicle-stimulating hormone
LH: Luteinizing hormone
FAI: Free androgen index
SHBG: Sex hormone-binding globin
FINS: Fasting serum insulin
FBG: Fasting blood glucose
HOMR-IR: Homeostasis model-insulin resistance
AMH: Anti-Müllerian hormone
INHB: Inhibin B
AR: Androgen receptor
Cx43: Connexins 43
EA: Electroacupuncture
ELISA: Enzyme-linked immunosorbent assay
IVF: *In vitro* fertilization
LE: Letrozole.
